# Screening for Fabry disease in unknown origin axonal polyneuropathy: to do or not to do, this is the question!

**DOI:** 10.1186/s13023-020-01501-w

**Published:** 2020-08-20

**Authors:** Eugenia Rota, Marina Grandis, Alessia Di Sapio, Elisabetta Ghiglione, Pietro Fiorentino, Alessandra Repetto, Claudia Giliberto, Chiara Gemelli, Nicola Morelli, Angelo Schenone, Dario Cocito

**Affiliations:** 1 Neurology Unit, ASL Alessandria (AL), Via E. Raggio 12, 15067 Novi Ligure, AL Italy; 2 Department of Neuroscience, Rehabilitation, Ophthalmology, Genetics and Maternal Infantile Sciences, University of Genoa, Genoa, Italy; 3 IRCCS Policlinico San Martino Hospital, Genoa, Italy; 4 Neurology Unit, Regina Montis Regalis Hospital, ASL CN1, Mondovì, CN Italy; 5 Neurology and Radiology Unit, Guglielmo da Saliceto Hospital, AUSLPC, Piacenza, PC Italy; 6 Istituti Clinici-Scientifici Maugeri, Torino, Italy

**Keywords:** Fabry disease, Neuropathy, Axonal, Polyneuropathy, Lysosomal disorder, Screening

## Abstract

Fabry disease (FD) is a systemic X-linked lysosomal disorder. A ‘peripheral nerve variant’ of FD has been hypothesized in subjects with neuropathy, without the early manifestations of the classic phenotype. A cohort of undiagnosed neuropathy patients with chronic polyneuropathy of undetermined aetiology and demyelinating neuropathy, unresponsive to immunomodulating treatment, were screened for FD. A total of 103 patients (64% males), were enrolled. No typical pathogenetic mutations for FD were identified. We are aware that the study sample was very small, but only a large, unfeasible theoretical sample size could demonstrate a statistically significant increased prevalence of FD in neuropathy patients, as peripheral neuropathy of undetermined cause is uncommon and there is a low prevalence of FD in the general population. Therefore, we are of the opinion that including tailored FD screening in the neuropathy diagnostic work-up, particularly when there are additional clinical characteristics, should be considered.

## Main text

Fabry disease (FD) is a systemic X-linked lysosomal disorder, caused by decreased activity of the enzyme α-galactosidase A (α-GAL A). Early diagnosis is pivotal, as enzyme replacement therapy is currently available for these patients. FD has a wide clinical spectrum, encompassing impaired peripheral nerve function, mostly of the small fibres, leading to an early-onset, usually painful, polyneuropathy.

Late-onset cardiac and renal variants of FD, due to missense mutations, with residual α-GAL A activity, have been described in subjects without the early, classical phenotype [1, 2]. Pathogenetic mutations of the α-GAL A gene (*GLA*) and genetic variants of unknown clinical significance (VUS) have been reported also in patients affected by small fibre neuropathy (SFN) of unknown aetiology, screened for FD, in a pilot study [3] and in a small case series [4]. Based on these data, a ‘*peripheral nerve variant*’ of FD has been hypothesized in subjects with neuropathy, without the early manifestations of the classic phenotype [4].

However, subsequent studies by other authors did not seem to support this hypothesis. Indeed, after FD screening, de Greef et al. [5] diagnosed no FD in 725 patients presenting with isolated SFN [5]. There was a reduced α-GAL A activity in 8 patients and a *GLA* gene variant was observed in 13, including one likely pathogenic variant in a female, although the diagnosis of FD could not be confirmed over time in this subject [5]. FD and Hereditary ATTR amyloidosis screening was done in another sample of 172 Nordic patients, affected by idiopathic small-fibre and mixed neuropathy [6]. A possible pathogenic variant, R118C, in the *GLA* gene was detected in a single patient, although clinical investigation identified no firm signs of FD [6]. Indeed, over the last few years, some *GLA* gene mutations previously classified as pathogenetic in screening studies (1995–2007) without confirmatory analyses, have recently been recognized as ‘benign/likely-benign’ variants [7].

On the other hand, newborn screening for lysosomal storage disorders (Pompe, Gaucher, Fabry disease, Mucopolysaccharidosis type I) by tandem mass spectrometry in North East Italy [8] revealed that the combined incidence of the 4 disorders was 1/4411 births and FD was the most frequent (1/8882). These data on FD prevalence in the general population raise the suspicion that the frequency of FD might have been underestimated in selected clinical samples.

Herein we report the results of an observational, cross-sectional, pilot study, carried out to investigate into the frequency of FD in a cohort of selected, undiagnosed neuropathy patients, with chronic polyneuropathy of undetermined aetiology [9] and with demyelinating neuropathy [10] unresponsive to immunomodulating treatment.

Patients (aged 18–80 years), referring to 5 Italian Neurology Units in 2 Northern Italian Regions (Piedmont: Torino, Casale Monferrato, Novi Ligure, Mondovì; Liguria: Genoa), with idiopathic axonal polyneuropathy (sensory or sensory-motor, painful or not, with or without comorbid SFN) were enrolled into the study, after obtaining written informed consent. Inclusion diagnostic criteria was chronic polyneuropathy of undetermined cause [9] or a previous diagnosis of chronic demyelinating neuropathy [10], unresponsive to immunomodulating treatment (at least three types of therapies). Exclusion criteria were: a positive family history of hereditary neuropathy, diabetes, alcohol abuse, dysthyroidism, monoclonal gammopathy, rheumatic and/or hemolymfoproliferative diseases, recent therapy with neurotoxic drugs.

All the patients were examined for α-GAL activity by Dried Blood Spot (DBS). Genetic analysis was carried out in all females and in males with a reduced α-GAL A activity (< 15.3 nmol/L/h) and/or in the low range, between 15.3 nmol/L/h and 20 nmol/L/h, of the normal limit interval (“pseudo-deficiency”). In line with the diagnostic protocol, whenever a mutation in the α-GAL A gene was identified, then the globotriaosylceramide (Gb3) levels were measured in urine and lyso-Gb3 in blood.

A total of 103 patients, 66 males (64%), average age 59 years, were enrolled (Table 1). Five of them also had SFN, confirmed by skin biopsy. The α-GAL A activity was normal in all males (average: 26.4 nmol/L/h). No typical pathogenetic mutations for FD were identified in the *GLA* gene in the study sample. Interestingly, a single genetic polymorphism was detected in a 67-year-old female patient with a SFN and diffuse neuropathic pain: c.937G > T (p.Asp313Tyr), previously classified as a VUS, more recently as a benign/likely benign exonic variant, according to Ncbi.Nlm.Nih.Gov database.

**Table 1:** Main clinical features

Total patients	103
Male (%)	66 (64%)
Age (years), mean (± SD)	60.3 (±13.1)
Axonal sensory-motor polyneuropathy (%)	48 (46.6%)
Axonal sensory polyneuropathy (%)	45 (43.7%)
Demyelinating neuropathy (%)	10 (9.7%)
Sensory symptoms (%)	85 (82.5%)
Motor symptoms (%)	51 (49.5%)
Small fiber neuropathy (%)	5 (4.8%)
α-GAL A activity in males (mean (± SD)	26.4 nmol/L/h (± 12.6)
Patients with systemic symptoms or signs of FD (%)	9 (8.7%)

Systemic signs or symptoms suggestive of FD were detected in 9 patients, 4 with previous myocardial infarction, 1 with slightly abnormal renal function (mild, i.e. not severe enough to be considered an exclusion criterion), 2 had cardiac arrhythmias, 2 had a history of cerebrovascular episodes.

Although the results of this pilot study apparently do not support the hypothesis of the late onset, ‘peripheral nerve variant’ of FD, these findings, though negative, cannot rule out such a novel hypothesis, due to the shortcomings of the study, mainly the small size of the sample, the high proportion of females enrolled and the diagnostic protocol.

Although DBS is a quite reliable and user-friendly tool to examine patients for α-GAL activity and to select male candidates for genetic analysis, we can not exclude that an alternative diagnostic flowchart, starting with the blood Gb3 level dosage before DBS and/or genetic evaluation, could have yielded different results. Indeed, according to recent studies [11], Gb3 levels are detectable in blood in all the male late-onset FD patients with residual α-GAL activity. Therefore, they seem to be a reliable biomarker to diagnose such cases with atypical presentations.

The main limitation of the study is the small sample size, similar to some previous ones. Indeed, if the frequency of FD in the general population is considered, then a much larger sample size than those previously reported on SFN and/or mixed neuropathy studies [3–6] would have to be enrolled to demonstrate a higher FD prevalence in idiopathic neuropathy.

Indeed, if we were to predict the same frequency of FD as that in patients with cryptogenic stroke (0.13%) in patients with undetermined neuropathy, then a theoretical sample size of: 31,853, 56,388 and 126,845 subjects respectively, for a relative inaccuracy accepted by 20 15, 10%, would be necessary to demonstrate this prevalence (0.13%). Moreover, there would be an unacceptable level of confidence, i.e. with a probability equal that of to 20% of a chance result.

Therefore, it is not conceivable to carry out a study, although collaborative, multi-center, reaching such a large sample size on neuropathy patients, considering that peripheral neuropathy of undetermined cause is quite uncommon, i.e. much rarer than stroke or renal failure. Consequently, it seems unlikely to find an Evidence-Based Medicine answer to the question: does the frequency of FD in patients suffering from neuropathy of unknown aetiology suffice to justify a screening in this clinical setting? Nevertheless, we believe that an individualized approach to each neuropathy patient may provide better clinical guidance. Indeed, we are of the opinion whether to test for FD or not should be carefully considered in the neuropathy diagnostic work-up, on an individual basis, mainly in the presence of additional clinical characteristics, indicative of FD. A new score for clinical suspicion of FD could include: 1) childhood and/or young adult onset; 2) comorbid personal and/or family history of cardio-cerebro-vascular disease; 3) renal failure; 4) typical skin lesions. All of which could be useful in the diagnostic assessment of patients with neuropathy of unknown aetiology to prompt FD screening.

Clinical experience may help the neurologist more than literature evidence in the decision-making process as to screening for FD undiagnosed neuropathy patients.

## Data Availability

The datasets used for the current study are available from the corresponding author on reasonable request.

## Abbreviations

FD: Fabry disease
α-GAL A: α-galactosidase A
GLA: α-GAL gene
VUS: genetic variants of unknown clinical significance
SFN: small fibre neuropathy
Gb3: globotriaosylceramide

## References

[1] von Scheidt W, Eng CM, Fitzmaurice TF, Erdmann E, Hübner G, Olsen EG, Christomanou H, Kandolf R, Bishop DF, Desnick RJ (1991). An atypical variant of Fabry's disease with manifestations confined to the myocardium. N Engl J Med.

[2] Nakao S, Kodama C, Takenaka T, Tanaka A, Yasumoto Y, Yoshida A (2003). Fabry disease: detection of undiagnosed hemodialysis patients and identification of a "renal variant" phenotype. Kidney Int.

[3] Tanislav C, Kaps M, Rolfs A, Böttcher T, Lackner K, Paschke E (2011). Frequency of Fabry disease in patients with small-fibre neuropathy of unknown aetiology: a pilot study. Eur J Neurol.

[4] Rota E, Morelli N, Terlizzi E, Iafelice I, Guidetti D (2014). Missing link: could the elusive Wartenberg's neuritis be a peripheral nerve variant of Fabry's disease?. Neurol India.

[5] de Greef BT, Hoeijmakers JG, Wolters EE, Smeets HJ, van den Wijngaard A, Merkies IS, Faber CG, Gerrits MM (2016). No Fabry disease in patients presenting with isolated small Fiber neuropathy. PLoS One.

[6] Samuelsson K, Radovic A, Press R, Auranen M, Ylikallio E, Tyynismaa H, KäRppä M, Veteläinen M, Peltola N, Mellgren SI, Mygland Å, Tallaksen C, Andersen H, Terkelsen AJ, Fontain F, Hietaharju A (2019). Screening for Fabry disease and HereditaryATTR amyloidosis in idiopathic small-fiber and mixed neuropathy. Muscle Nerve.

[7] Doheny D, Srinivasan R, Pagant S, Chen B, Yasuda M, Desnick RJ (2018). Fabry disease: prevalence of affected males and heterozygotes with pathogenic GLA mutations identified by screening renal, cardiac and stroke clinics, 1995-2017. J Med Genet.

[8] Burlina AB, Polo G, Salviati L, Duro G, Zizzo C, Dardis A, Bembi B, Cazzorla C, Rubert L, Zordan R, Desnick RJ, Burlina AP (2018). Newborn screening for lysosomal storage disorders by tandem mass spectrometry in north East Italy. J Inherit Metab Dis.

[9] McLeod JG, Tuck RR, Pollard JD, Cameron J, Walsh JC (1984). Chronic polyneuropathy of undetermined cause. J Neurol Neurosurg Psychiatry.

[10] European Federation of Neurological Societies/Peripheral Nerve Society Guideline on management of chronic inflammatory demyelinating polyradiculoneuropathy: report of a joint task force of the European Federation of Neurological Societies and the Peripheral Nerve Society-First Revision. Joint Task Force of the EFNS and the PNS. J Peripher Nerv Syst. 2010;15(1):1–9. 10.1111/j.1529-8027.2010.00245.x 20433600

[11] Duro G, Zizzo C, Cammarata G, Burlina A, Burlina A, Polo G, Scalia S, Oliveri R, Sciarrino S, Francofonte D, Alessandro R, Pisani A, Palladino G, Napoletano R, Tenuta M, Masarone D, Limongelli G, Riccio E, Frustaci A, Chimenti C, Ferri C, Pieruzzi F, Pieroni M, Spada M, Castana C, Caserta M, Monte I, Rodolico MS, Feriozzi S, Battaglia Y, Amico L, Losi MA, Autore C, Lombardi M, Zoccali C, Testa A, Postorino M, Mignani R, Zachara E, Giordano A, Colomba P. Mutations in the GLA Gene and LysoGb3: Is It Really Anderson-Fabry Disease? Int J Mol Sci. 2018;19(12):3726. 10.3390/ijms19123726 PMC6320967 30477121

